# Healthy Nordic diet and associations with plasma concentrations of metabolites in the choline oxidation pathway: a cross-sectional study from Northern Sweden

**DOI:** 10.1186/s12937-023-00853-w

**Published:** 2023-05-17

**Authors:** André Hesselink, Anna Winkvist, Bernt Lindahl, Per M. Ueland, Jörn Schneede, Ingegerd Johansson, Therese Karlsson

**Affiliations:** 1 Department of Internal Medicine and Clinical Nutrition, University of Gothenburg, Box 459, 405 30 Gothenburg, Sweden; 2 Department of Public Health and Clinical Medicine, Sustainable Health, Umeå University, Umeå, Sweden; 3 Department of Clinical Science, University of Bergen, Bergen, Norway; 4 Bevital AS, Bergen, Norway; 5 Department of Clinical Pharmacology, Pharmacology and Clinical Neurosciences, Umeå University, Umeå, Sweden; 6 Department of Odontology, Umeå University, Umeå, Sweden; 7 Department of Life Sciences, Chalmers University of Technology, Gothenburg, Sweden

**Keywords:** Healthy Nordic diet, Healthy Nordic Food Index, Baltic Sea Diet Score, One-carbon metabolism, Choline oxidation pathway, Västerbotten Intervention Programme

## Abstract

**Background:**

The choline oxidation pathway and metabolites involved have been linked to diseases including cardiovascular disease, type 2 diabetes and cancer. A healthy Nordic diet is a recently defined dietary pattern associated with decreased risk for these diseases. Our aim was to explore associations between adherence to a healthy Nordic diet and plasma concentrations of metabolites of the choline oxidation pathway.

**Methods:**

The Healthy Nordic Food Index (HNFI) and Baltic Sea Diet Score (BSDS) were applied to cross-sectional data (*n* = 969) from the Västerbotten Intervention Programme in Northern Sweden to score adherence to a healthy Nordic diet. Data included responses to a dietary questionnaire and blood sample analyses (1991–2008). Associations of diet scores with plasma concentrations of metabolites of the choline oxidation pathway and total homocysteine (tHcy), seven metabolites in total, were evaluated with linear regression, adjusting for age, BMI, education and physical activity.

**Results:**

HNFI scores showed linear relationships with plasma choline (β = 0.11), betaine (β = 0.46), serine (β = 0.98) and tHcy (β =  − 0.38), and BSDS scores with betaine (β = 0.13) and tHcy (β =  − 0.13); unstandardized beta coefficients, all significant at *P* < 0.05. The regression models predicted changes in plasma metabolite concentrations (± 1 SD changes in diet score) in the range of 1–5% for choline, betaine, serine and tHcy. No other statistically significant associations were observed.

**Conclusions:**

A healthy Nordic diet was associated with plasma concentrations of several metabolites of the choline oxidation pathway. Although relationships were statistically significant, effect sizes were moderate. Further research is warranted to explore the underlying mechanisms and associations with health outcomes.

**Supplementary Information:**

The online version contains supplementary material available at 10.1186/s12937-023-00853-w.

## Background

**Fig. 1:**
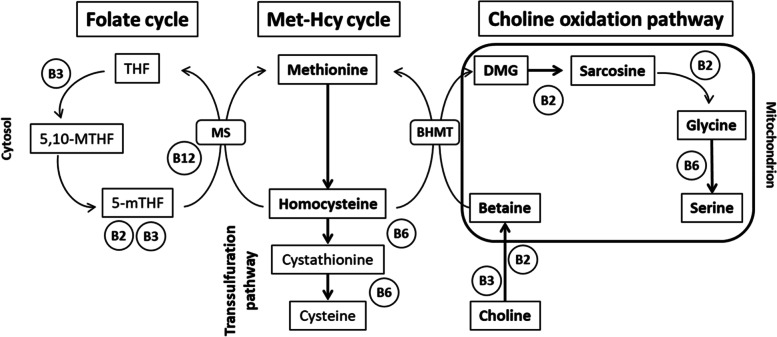
One-carbon metabolic processes: the folate cycle, methionine-homocysteine cycle and choline oxidation pathway. Legend: Abbreviations: Met-Hcy, methionine-homocysteine; DMG, dimethylglycine; THF, tetrahydrofolate; 5-mTHF, methyltetrahydrofolate; 5,10-MTHF, methylenetetrahydrofolate; MS, methionine synthase; BHMT, betaine-homocysteine methyltransferase; B2, riboflavin; B3, niacin; B6, pyridoxine; B12, cobalamin. Adapted from Lysne et al. Nutrients 2016, 8, 26; https://doi.org/10.3390/nu8010026. Licensed under CC BY 4.0 (https://creativecommons.org/licenses/by/4.0/)

The choline oxidation pathway and its related metabolites have been linked to the development of cardiovascular disease (CVD), type 2 diabetes, cancer and all-cause mortality [1–4]. This pathway is part of a larger set of one-carbon metabolic processes including the methionine-homocysteine and folate cycles (Fig. 1). These metabolic processes play a central role in the biosynthesis of a wide range of molecules, genomic maintenance, genetic expression and cellular redox homeostasis [5]. Some previous studies, but not all, have shown positive associations between blood concentrations of choline, dimethylglycine (DMG) and homocysteine; and cardiometabolic risk [1–3, 6, 7]. Betaine, on the other hand, has shown inverse associations with CVD risk as well as with colorectal cancer [2, 4].

Food and nutrient intake can directly affect circulating concentrations of metabolites involved in the choline oxidation pathway. Foods rich in choline such as eggs have shown a positive association with concentrations of choline [8, 9]. Similarly, whole grains, high-fiber bread and other complex carbohydrates, all rich in betaine, have been positively linked to concentrations of betaine [9, 10]. The diet’s effect on concentrations of homocysteine have also been studied extensively with fish, egg, whole-grain and vegetable intake as well as many B-vitamins showing inverse associations with homocysteine concentrations [11–13].

A healthy Nordic diet is a recently defined dietary pattern based on traditional foods originating from the Nordic region with documented health benefits. Typical foods include apples, pears, berries, root vegetables, cabbages, legumes, fatty fish and whole grains [14]. Additional health-promoting foods and eating patterns such as low-fat dairy products and low consumption of red and processed meat have also been included in the definition of a healthy Nordic diet [15]. In terms of health benefits, a healthy Nordic diet has been associated with improved blood lipid profile, insulin sensitivity and blood pressure, decreased abdominal obesity and a lower rate of all-cause mortality [14, 16, 17]. Researchers have created dietary indexes to measure adherence to a healthy Nordic diet, two of the more widely adopted indexes being the Healthy Nordic Food Index (HNFI) and the Baltic Sea Diet Score (BSDS) [15, 16].

Few studies have investigated the associations between a healthy Nordic diet and circulating metabolite concentrations with choline oxidation as a main focus [18–20]. The aim of our study was to explore potential associations between adherence to a healthy Nordic diet, as measured by the HNFI and BSDS, and blood plasma concentrations of metabolites involved in choline oxidation. In addition, we evaluated intakes of choline and betaine in relation to adherence to a healthy Nordic diet, which could potentially help explain associations between the dietary indexes and circulating metabolites.

## Methods

### Study population

The Västerbotten Intervention Programme (VIP) is a primary care-based initiative launched in 1985 due to a higher-than-expected mortality rate from CVD in the Västerbotten region of Northern Sweden. To this day, residents are invited to receive a voluntary health examination when they turn 40, 50 and 60 years old. From 1990–2018, there have been over 184,000 visits in the program, averaging around 6,000 health examinations per year [21]. Health examinations include anthropometric measurements (weight, height, BMI), blood pressure measurement, an oral glucose tolerance test and blood tests for lipid profiles and plasma glucose concentrations. In connection with blood sampling as part of the health examination, participants are asked if they would like to donate additional blood for future research purposes. Participants are also asked to fill in an extensive questionnaire covering overall health, socio-economic and psychosocial well-being, quality of life and other aspects of life. The final component of the health examination is a self-administered food frequency questionnaire (FFQ) to estimate dietary intake [22, 23].

The dataset for the present study was an extract from the main VIP database initially compiled for a planned nested case–control study exploring associations between folate intake, folate status and the incidence of colorectal cancer. Details of this dataset are described in a separate study on one-carbon metabolite ratios and colorectal cancer risk [24]. Exclusion criteria for the initial case–control study included previous cancer diagnosis other than non-melanoma skin cancer, insufficient blood plasma volume for analysis, primary tumor located outside the colorectum (cases only), plasma samples prioritized for other studies, serious infectious diseases and no matching control available. The median follow-up time to cancer diagnosis was 8.2 years [24].

The starting dataset for the present study consisted of 383 cases and 737 controls for a total of 1,120 observations with health examinations ranging from 1991 to 2008. Participants with any of the following attributes were subsequently excluded: height less than 130 cm or greater than 210 cm (*n* = 7), weight less than 35 kg (*n* = 7), BMI less than 15 kg/m^2^ (*n* = 12) or food intake level within the 1^st^ or 99^th^ percentiles (*n* = 101). Non-reporters with respect to the FFQ were also excluded (*n* = 124), defined as either leaving more than 10% of the questions unanswered or a missing answer for any of the three portion size questions. These exclusion criteria are in line with method recommendations from the Unit for Biobank Research, Umeå University, Sweden [25], as well as with previous publications from the VIP.

Three duplicate participant IDs were identified due to a second blood sample analysis, and observations connected to the second blood sampling were excluded. After exclusions for extremes, non-reporters and duplicates (*n* = 151), 969 observations were included in the final data analyses.

Physical activity was based on participants’ responses to a question on how often they had trained or exercised in the past three months, from 1 = never and up to 5 = more than three times per week. Responses were re-coded for the purposes of this study to low (= 1, 2), medium (= 3, 4) or high (= 5). Similarly, school education was categorized as base level, i.e., up to high school (responses 1–6), high school (responses 7 and 8) and university (response 9). With regards to smoking status, participants were categorized as 1 = present smoker, 2 = ex-smoker or 3 = never. In this study, smoking status was re-coded to present smoking (= 1) and non-smokers (= 2, 3).

Blood samples were collected in the morning with 91% of subjects having fasted more than eight hours and 98% more than four hours Samples were collected in EDTA (ethylenediamine tetra-acetic acid) tubes, separated into plasma, erythrocyte fractions and buffy coat, and frozen within one hour of collection [24]. All samples were analyzed at Bevital A/S (http://www.bevital.no), Bergen, Norway in 2011. Plasma concentrations of choline, betaine and DMG were measured using liquid chromatography-tandem mass spectrometry [26]. Plasma concentrations of total homocysteine (tHcy), sarcosine, glycine and serine were measured with gas chromatography-tandem mass spectrometry [27, 28]. Biomarkers in the one-carbon metabolism have shown to have adequate stability and reproducibility [29]. All plasma concentrations are reported in µmol/L.

### Dietary assessment and intake estimation

The FFQ used to collect dietary intake data existed in two main versions during the observation period (1991–2008): the first questionnaire with 84 food items and a second, shorter version with 64 items where previous questions had been combined. In later versions, two additional questions were added for consumption of water and eggs. Three additional questions were related to portion sizes. Questions addressing specific food items were answered using a 9-level frequency scale: “never”, “a few times per year”, up to a maximum frequency response of “4 times per day or more”. Food items included various types of fat (bread spreads, cooking oils), dairy products, fruits and vegetables, soft drinks, sugar-containing snacks, bread, cereals, rice, pasta, potatoes, meat, fish, salty snacks, coffee, tea, juice and alcoholic beverages. Additional information regarding the FFQ can be found at https://www.umu.se/en/biobank-research-unit/.

The 84-item FFQ has been validated against repeated 24-h dietary recalls (24-HDR) and biomarkers for total energy intake as well as the intake of several nutrients and food items [23, 30, 31]. Daily energy and most nutrient intakes were similar for the FFQ and 24-HDR; however, the FFQ recorded higher values for fiber and lower values for cholesterol. Moderately higher intake frequencies were recorded by the FFQ for fruits, vegetables, bread and cereals, potatoes/rice/pasta and dairy products, and lower intake frequencies for fish, meat, sweet snacks and alcohol. Overall, the FFQ has a validity level in line with FFQ measurements of other prospective cohort studies [23].

Daily energy and nutrient intakes were calculated for the major macronutrients, types of fat, vitamins and minerals. These were calculated based on the FFQ responses. All reported frequencies were converted to intakes per day, and then multiplied by portion sizes and energy or nutrient content from a food composition database maintained by the Swedish National Food Administration (https://www7.slv.se/SokNaringsinnehall/). Portion sizes were based on FFQ answers or standard portion sizes for sex and age according to a previous national survey [23].

The U.S. Department of Agriculture (USDA) Database for the Choline Content of Common Foods, release 2 [32] was used to calculate the daily intakes of total choline, individual forms of choline and betaine. A few later updates of the database were included from 2015, accessible via https://fdc.nal.usda.gov/ and one additional source for the choline content in cream [33]. Total choline is calculated as the sum of free choline, glycerophosphocholine (GPC), phosphocholine (PCho), phosphatidylcholine (PtdCho) and sphingomyelin (SM). If a food item from the FFQ was not available in the USDA database, then a nutritionally equivalent food was substituted for the calculations. Additionally, dishes from the FFQ that were unavailable were broken down to their ingredients for the estimate of choline and betaine content.

### The healthy Nordic dietary indexes

Both the HNFI and BSDS were used to measure participants’ adherence to a healthy Nordic diet. The purpose of using two different indexes was to assess if the study results could be affected by how one defined a healthy Nordic diet. However, this was not a primary aim of the study.

The HNFI was adapted from Olsen et al. [16] and included six food sub-categories: fish, cabbages, whole grain rye, whole grain oats, apples and pears, and root vegetables. For each sub-category, a score of 0 or 1 was given based on whether the participant was below or above the relevant sex-specific median for the respective sub-category. A participant’s total score for the HNFI could range from 0 to 6 points.

The BSDS was based on Kanerva et al. [15] and consisted of nine sub-categories: fruits and berries, vegetables, cereals, low-fat dairy, fish, meat products, total fat in energy percent (E%), fat ratio (PUFAs to SFAs + trans-fatty acids) and alcohol. Scoring for the first eight sub-categories was based on sex-specific quartiles with participants receiving 0 to 3 points per sub-category: 1^st^ quartile = 0, 2^nd^ quartile = 1, 3^rd^ quartile = 2 and 4^th^ quartile = 3. Two exceptions were meat products and total fat consumption, which used the same scoring system but on an inverse order scale, i.e., 1^st^ quartile = 3, 2^nd^ quartile = 2, and so on. The ninth sub-category, alcohol consumption, was scored based on sex-specific cut-offs for moderate consumption as defined by the Nordic Nutrition Recommendations 2012 [34], 10 and 20 g per day for women and men, respectively. Participants received 1 point for daily consumption under the cut-off or 0 points above. A participant’s total score for the BSDS could range from 0 to 25 points.

The relevant FFQ questions were mapped to twelve of the food sub-categories defined by the HNFI and BSDS. The remaining three sub-categories from the BSDS (total fat, fat ratio and alcohol) were based on calculated nutrient values from FFQ responses. Separate mappings were required for each of the FFQ versions (long and short versions) to account for inconsistencies between the two versions. The detailed mappings can be found in the Online Resource 1. Participants were then grouped by FFQ version and sex, and each group scored separately according to median, quartiles or reference cut-offs as described above. All intakes, initially in grams per day (g/d), were energy-adjusted by the density method to g/d per 1,000 kcal before calculation of sex-specific medians and quartiles [35]. Three exceptions to this were total fat, which was converted to E%, fat ratio and alcohol. Once the scoring was complete, groups were recombined for the analysis.

### Statistical analysis

All computations were run in IBM SPSS Statistics version 27. All tests with regards to the correlation coefficients and regression models were two-tailed and *P* < 0.05 was considered statistically significant.

Distributions of the two diet index scores and plasma metabolite concentrations were inspected for normality with the help of descriptive statistics and histogram plots. Linear correlation for each pair of variables (independent vs. dependent) was inspected visually via scatterplot diagrams. Two observations were classified as outliers and excluded from the analyses: one participant with plasma choline greater than 20 µmol/L and a second with plasma betaine greater than 200 µmol/L.

Pearson correlation coefficients were used to evaluate linear correlations between dietary intake of choline and betaine (g/d per 1,000 kcal) and the diet index scores; and between the diet index scores and plasma concentrations of the metabolites of interest. Scatterplot diagrams did not suggest non-linear relationships between the variables. Multivariable linear regression models were fitted separately for each diet index score variable (independent variables) against each of the metabolites of interest (dependent variables), 14 regression models in total. Each regression model was adjusted for six potential confounding variables, separately: age, sex, BMI, smoking status, physical activity and education level. Confounding variables having more than a 10% effect on the metabolite’s beta coefficient in any of the models were included in the final adjusted models for all metabolites. The final confounding variables included were age, BMI, physical activity level and education level.

## Results

### Baseline characteristics and nutrient intake

Baseline characteristics and nutrient intakes of the study population segmented by index score are shown in Tables 1, 2, 3 and 4. No material differences with respect to baseline characteristics and nutrient intakes were observed between the two Nordic dietary indexes. The average age of all participants was 54 ± 7.4 years (mean ± SD). Participants in the higher score segments were slightly older. Similarly, upward trends across score segments could be seen for school education (especially university), BMI and physical activity (Tables 1 and 2).

**Table 1:** Subject characteristics segmented by Healthy Nordic Food Index (HNFI) score

		**Index score segments**		
**Characteristic**^**a**^	**All**	**1**	**2**	**3**
HNFI scores	3.0 ± 1.5	0–2	3–4	5–6
Observations, n	969	386	411	172
Age, years	54 ± 7.4	53 ± 8.4	54 ± 6.7	56 ± 6.0
Sex (% female)	46	45	47	47
School education (%)				
Base level	66	72	63	58
High school	15	16	14	15
University	19	12	22	27
BMI, kg/m^2^	26.0 ± 3.7	25.9 ± 3.7	25.8 ± 3.5	26.9 ± 4.1
Physical activity (%)				
Low	77	86	72	64
Medium	20	11	24	29
High	4	2	4	7
Present smoking (%)	18	17	19	19
Metabolites (µmol/L)				
Choline	8.9 ± 1.6	8.7 ± 1.5	9.0 ± 1.6	9.2 ± 1.8
Betaine	31.0 ± 7.2	30.1 ± 7.2	31.6 ± 7.5	32.8 ± 15.4
tHcy	10.5 ± 4.7	11.2 ± 6.0	10.2 ± 3.9	9.9 ± 3.0
DMG	3.8 ± 1.8	3.8 ± 1.6	3.8 ± 2.0	3.9 ± 1.7
Sarcosine	1.9 ± 1.1	1.9 ± 1.1	1.8 ± 1.1	2.1 ± 1.2
Glycine	244 ± 73	242 ± 70	245 ± 73	245 ± 81
Serine	110 ± 19	110 ± 19	110 ± 19	112 ± 20

*Abbreviations*: *DMG* Dimethylglycine, *HNFI* Healthy Nordic Food Index, *tHcy* total homocysteine

^a^Values presented as means ± SD or percentages

**Table 2:** Subject characteristics segmented by Baltic Sea Diet Score (BSDS)

		**Index score segments**				
**Characteristic**^**a**^	**All**	**1**	**2**	**3**	**4**	**5**
BSDS scores	12.9 ± 4.1	0–8	9–11	12–13	14–16	17–24
Observations, n	969	131	240	170	226	202
Age, years	54 ± 7.4	52 ± 8.5	52 ± 8.4	54 ± 7.2	55 ± 6.2	56 ± 6.0
Sex (% female)	46	49	44	48	43	49
School education (%)						
Base level	66	74	68	60	67	62
High school	15	17	21	14	12	12
University	19	9	12	26	21	27
BMI, kg/m^2^	26.0 ± 3.7	25.6 ± 3.9	26.0 ± 4.0	25.9 ± 3.6	26.2 ± 3.3	26.4 ± 4.0
Physical activity (%)						
Low	77	88	82	79	75	63
Medium	20	11	16	17	22	31
High	4	2	3	5	4	6
Smoking (%)	18	14	17	19	23	16
Metabolites (µmol/L)						
Choline	8.9 ± 1.6	8.6 ± 1.6	8.9 ± 1.7	8.8 ± 1.5	8.9 ± 1.6	9.2 ± 1.7
Betaine	31.0 ± 7.2	29.6 ± 6.4	31.3 ± 7.2	30.2 ± 7.0	31.1 ± 7.5	33.1 ± 14.9
tHcy	10.5 ± 4.7	10.6 ± 3.3	11.3 ± 6.0	10.3 ± 4.2	10.7 ± 5.7	9.6 ± 2.3
DMG	3.8 ± 1.8	3.8 ± 2.0	3.9 ± 1.8	3.8 ± 2.2	3.7 ± 1.5	3.8 ± 1.5
Sarcosine	1.9 ± 1.1	1.8 ± 1.0	1.9 ± 1.1	1.9 ± 1.2	1.9 ± 1.2	1.9 ± 1.1
Glycine	244 ± 73	235 ± 60	241 ± 69	253 ± 76	243 ± 77	247 ± 77
Serine	110 ± 19	108 ± 17	111 ± 20	111 ± 19	110 ± 19	111 ± 20

*Abbreviations*: *BSDS* Baltic Sea Diet Score, *DMG* Dimethylglycine, *tHcy* total homocysteine

^a^Values presented as means ± SD or percentages

**Table 3:** Dietary intake segmented by Healthy Nordic Food Index (HNFI) score (*n* = 969)

	**HNFI segments**		
**Nutrient**^**a**^	**1**	**2**	**3**
HNFI scores	0–2	3–4	5–6
Observations, n	386	411	172
Energy intake, kcal/d	1,810 ± 620	1,779 ± 638	1,707 ± 533
Protein, g/d	63 ± 22	65 ± 24	63 ± 20
Animal protein, g/d	45 ± 18	45 ± 19	42 ± 16
Plant-based protein, g/d	18 ± 7	20 ± 8	21 ± 7
Protein, E%	14 ± 2	15 ± 2	15 ± 2
Carbohydrates, E%	49 ± 6	51 ± 6	53 ± 5
Total fat, E%	35 ± 6	33 ± 6	30 ± 5
SFAs, E%	15 ± 3	14 ± 3	12 ± 3
PUFAs, E%	4.8 ± 1.6	4.7 ± 1.4	4.9 ± 1.8
Fiber, g/d	17 ± 7	21 ± 8	24 ± 7
Alcohol, E%	1.6 ± 1.9	1.6 ± 1.9	1.7 ± 2.0
Total choline, mg/d	239 ± 79	250 ± 80	257 ± 75
Betaine, mg/d	147 ± 60	169 ± 75	179 ± 69
B-vitamins			
Folate, µg/d	198 ± 72	238 ± 82	278 ± 97
Cobalamin (B12), µg/d	4.6 ± 2.1	4.7 ± 2.3	4.9 ± 2.4
Riboflavin (B2), µg/d	1.4 ± 0.5	1.4 ± 0.5	1.4 ± 0.5
Vitamin B6, mg/d	1.9 ± 0.8	2.0 ± 0.7	2.1 ± 0.7

*Abbreviations*: *HNFI* Healthy Nordic Food Index, *PUFA* Polyunsaturated Fatty Acid, *SFA* Saturated Fatty Acid

^a^Values presented as means ± SD

**Table 4:** Dietary intake segmented by Baltic Sea Diet Score (*n* = 969)

	**BSDS segments**				
**Nutrient**^**a**^	**1**	**2**	**3**	**4**	**5**
BSDS scores	0–8	9–11	12–13	14–16	17–24
Observations, n	131	240	170	226	202
Energy intake (kcal/d)	1,856 ± 719	1,854 ± 649	1,697 ± 633	1,789 ± 547	1,696 ± 534
Protein, g/d	65 ± 28	65 ± 24	61 ± 23	64 ± 20	63 ± 20
Animal protein, g/d	48 ± 22	46 ± 19	43 ± 18	44 ± 16	42 ± 15
Plant-based protein, g/d	17 ± 7	19 ± 8	18 ± 7	20 ± 7	22 ± 7
Protein, E%	14 ± 3	14 ± 2	15 ± 2	14 ± 2	15 ± 2
Carbohydrates, E%	45 ± 6	49 ± 5	50 ± 6	52 ± 6	55 ± 5
Total fat, E%	39 ± 5	35 ± 5	33 ± 5	31 ± 5	28 ± 4
SFAs, E%	17 ± 3	15 ± 2	14 ± 2	13 ± 3	11 ± 2
PUFAs, E%	4.9 ± 1.5	4.8 ± 1.7	4.7 ± 1.5	4.7 ± 1.5	4.7 ± 1.6
Fiber, g/d	15 ± 6	18 ± 7	18 ± 7	22 ± 7	24 ± 8
Alcohol, E%	1.5 ± 1.9	1.6 ± 1.9	1.8 ± 2.1	1.6 ± 1.8	1.6 ± 1.8
Total choline, mg/d	241 ± 93	248 ± 81	236 ± 81	250 ± 71	255 ± 72
Betaine, mg/d	141 ± 60	157 ± 68	151 ± 64	169 ± 66	184 ± 78
B-vitamins					
Folate, µg/d	181 ± 70	209 ± 68	218 ± 81	241 ± 75	280 ± 102
Cobalamin (B12), µg/d	4.6 ± 2.1	4.8 ± 2.1	4.5 ± 2.2	4.8 ± 2.3	4.7 ± 2.3
Riboflavin (B2), µg/d	1.3 ± 0.5	1.4 ± 0.5	1.4 ± 0.5	1.5 ± 0.5	1.5 ± 0.5
Vitamin B6, mg/d	1.8 ± 0.7	1.9 ± 0.7	1.9 ± 0.8	2.0 ± 0.7	2.0 ± 0.7

*BSDS* Baltic Sea Diet Score, *PUFA* Polyunsaturated Fatty Acid, *SFA* Saturated Fatty Acid

^a^Values presented as means ± SD

Both indexes also showed similar trends in baseline nutrient intakes across score segments (Tables 3 and 4). Total daily energy intake decreased across score segments, 1,810 ± 620 and 1,707 ± 533 kcal/d for HNFI segments 1 and 3, respectively; and 1,856 ± 719 and 1,696 ± 534 kcal/d for BSDS segments 1 and 5, respectively. Higher index scores reflected a shift from animal to plant-based protein. Carbohydrates as a % of total energy intake (E%) increased consistently across score segments, with the opposite being true for total fat intake (E%). SFAs (E%) decreased with higher index scores; however, intake of PUFAs (E%) remained relatively unchanged.

Fiber intake trended higher across index score segments as well as intakes of total choline and betaine. Similarly, folate intake showed a marked increase with daily intake at 198 ± 72 and 278 ± 97 µg/d for HNFI segments 1 and 3, respectively; and 181 ± 70 and 280 ± 102 µg/d for segments 1 and 5, respectively. Finally, intakes of other B-vitamins showed slightly upward trends across the HNFI and BSDS score segments. Food intakes across HNFI and BSDS index scores are shown in Supplementary Tables 1 and 2.

### Correlation analysis: Nordic dietary indexes vs. choline, betaine intake

**Fig. 2:**
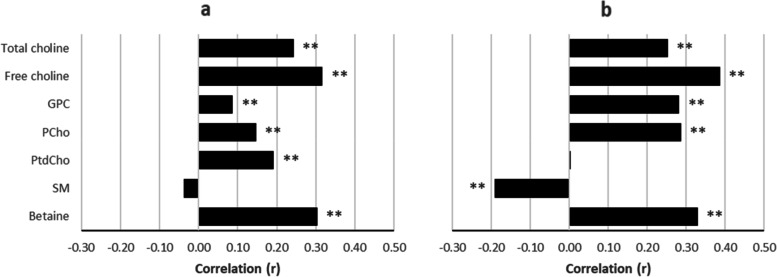
Pearson correlation coefficients, Nordic dietary indexes vs. intakes of total choline, choline species and betaine. Legend: **a** Healthy Nordic Food Index. **b** Baltic Sea Diet Score. Dietary intake in g/d * 1000 kcal.^−1^ Abbreviations: GPC, glycerophosphocholine; PCho, phosphocholine; PtdCho, phosphatidylcholine; SM, sphingomyelin. ** *P* < 0.01

Pearson correlation coefficients (*r*) between dietary intake of choline and betaine, and the two healthy Nordic diet scores are presented in Fig. 2. Betaine and all forms of choline except SM showed a positive linear relationship with HNFI score at a significance level of *P* < 0.01. The highest correlation with the HNFI was observed with betaine and free choline, *r* = 0.30 and 0.32, respectively. All nutrients except PtdCho showed significant correlations (*P* < 0.01) with BSDS scores. Again, betaine and free choline had the highest correlations with the BSDS, *r* = 0.33 and 0.39, respectively. However, SM demonstrated a negative linear relationship with *r* =  − 0.19. Correlation coefficients between dietary choline and plasma concentrations of metabolites are shown in Supplementary Fig. 1.

### Correlation analysis: Nordic dietary indexes vs. circulating metabolites

**Fig. 3:**
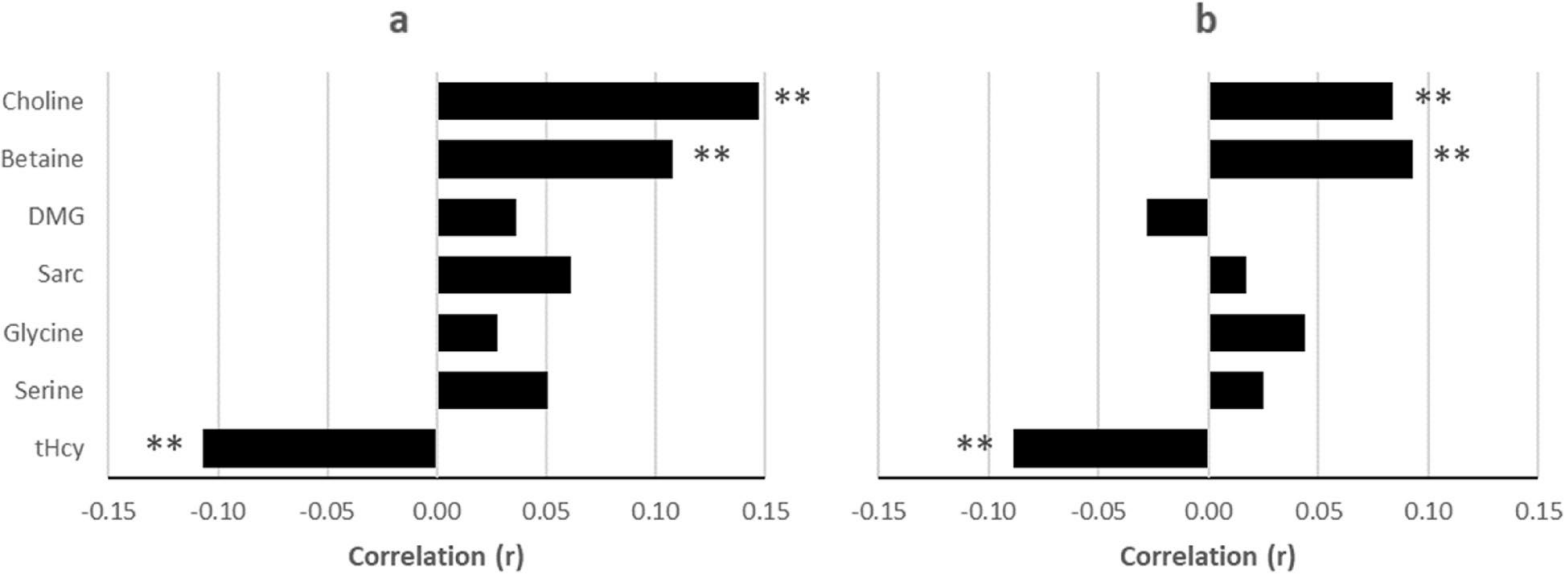
Pearson correlation coefficients. Nordic dietary indexes vs. plasma metabolites. Legend: **a** Healthy Nordic Food Index. **b** Baltic Sea Diet Score. Abbreviations: DMG, dimethylglycine; Sarc, sarcosine; tHcy, total homocysteine. ** *P* < 0.01

Pearson correlation coefficients for diet index scores and plasma concentrations of metabolites are shown in Fig. 3. Positive linear relationships (P < 0.01) were observed between HNFI score and the metabolites choline and betaine, *r* = 0.15 and 0.11, respectively. tHcy showed a negative correlation with HNFI score, *r* =  − 0.11 and *P* < 0.01. Similar results were observed vs. BSDS index scores; however, linear relationships were slightly attenuated. Correlation between the two diet score indexes was r = 0.73 (*P* < 0.001). Correlation coefficients between the separate food components of the HNFI and the BSDS, and plasma concentrations of metabolites are shown in Supplementary Figs. 2 and 3.

### Multivariable linear regression: crude and adjusted models

Results from multivariable linear regression analyses are listed in Tables 5 and 6. Choline and betaine showed positive associations with HNFI score before adjustment for confounders, β = 0.15 (*P* < 0.001) and 0.50 (*P* < 0.001), respectively. tHcy was inversely related to HNFI score with β =  − 0.33 (*P* < 0.001). The positive associations were attenuated after adjusting for confounding variables but remained significant. In contrast, this adjustment strengthened the negative association of tHcy with HNFI score from − 0.33 to − 0.38 (*P* < 0.001**)**. After adjustment for confounders, serine showed a positive association with HNFI score, β = 0.98 (*P* = 0.019). Sensitivity analysis stratifying cases and controls did not materially change the results (data not shown).

**Table 5:** Results from multivariable linear regression of Healthy Nordic Food Index (HNFI) score vs. plasma concentrations of metabolites

	**Crude**			**Adjusted**^**a**^		
**Metabolite**	β	SE	P	β	SE	P
Choline	0.15	0.03	< 0.001	0.11	0.03	0.002
Betaine	0.50	0.15	0.001	0.46	0.16	0.004
tHcy	- 0.33	0.10	0.001	- 0.38	0.11	< 0.001
DMG	0.04	0.04	0.26	0.03	0.04	0.52
Sarcosine	0.04	0.02	0.06	0.01	0.02	0.70
Glycine	1.30	1.53	0.39	1.44	1.61	0.37
Serine	0.64	0.40	0.11	0.98	0.42	0.019

*Abbreviations*: *DMG* Dimethylglycine, *tHcy* total homocysteine

^a^Adjusted for age, BMI, physical activity and education level

**Table 6:** Results from multivariable linear regression of Baltic Sea Diet Score (BSDS) score vs. plasma concentrations of metabolites

	**Crude**			**Adjusted**^**a**^		
**Metabolite**	β	SE	P	β	SE	P
Choline	0.03	0.01	0.015	0.02	0.01	0.26
Betaine	0.15	0.06	0.007	0.13	0.06	0.026
tHcy	- 0.12	0.04	0.002	- 0.13	0.04	0.001
DMG	- 0.01	0.01	0.43	- 0.02	0.01	0.17
Sarcosine	0.01	0.01	0.46	- 0.01	0.01	0.4´7
Glycine	0.66	0.58	0.25	0.69	0.60	0.25
Serine	0.15	0.15	0.34	0.27	0.16	0.08

*Abbreviations*: *DMG* Dimethylglycine, *tHcy* total homocysteine

^a^Adjusted for age, BMI, physical activity and education level

Similar results were obtained from regression analysis with BSDS score as the independent variable. Again, choline, betaine and tHcy showed significant associations vs. the index score before adjustment. Values of beta coefficients were lower compared to regressions with HNFI, but it should be noted that the range of the BSDS scale is roughly four times larger than the HNFI. Beta coefficient values were attenuated after adjustment for confounding except in the case of tHcy. Only betaine and tHcy remained statistically significant at the *P* < 0.05 level in the adjusted models. Sensitivity analysis stratifying cases and controls did not materially change the results (data not shown).

Predicted changes in plasma metabolite concentrations relative to sample population means were assessed by applying a one standard deviation increase in diet score (HNFI: + 1.5, BSDS: + 4.1) to the above regression equations. For the HNFI, regression models predicted increases in metabolite concentrations for choline (+ 1.8%; 95% CI: 0.7%, 2.9%), betaine (+ 2.2%; 95% CI: 0.7%, 3.7%) and serine (+ 1.3%; 95% CI: 0.2%, 2.4%), and a decrease for tHcy (-5.4%; 95% CI: -8.3%, -2.4%). For the BSDS, regression models predicted an increase for betaine (+ 1.7%; 95% CI: 0.2%, 3.3%) and a decrease for tHcy (-5.2%; 95% CI: -8.1%, -2.2%).

## Discussion

In this cross-sectional study of a population in Northern Sweden, we identified four metabolites of the choline oxidation pathway that were associated with two healthy Nordic dietary indexes using a multivariable linear regression analysis. Choline, betaine and serine showed positive linear associations with the indexes while tHcy showed negative associations. In addition, we found statistically significant associations between choline and betaine intake and the dietary indexes.

### Associations of the dietary indexes and the choline oxidation pathway

Correlations between the dietary indexes and the metabolites were in the lower range (*r* =  − 0.11 to 0.15), which is in line with previously reported results of healthy eating indexes and concentrations of other metabolites [19, 20]. This observation is not surprising since one-carbon metabolism is such a central part of human metabolism and affected by many factors other than diet. Nonetheless, the existing literature on the effects of diet on one-carbon metabolites can help explain these modest but statistically significant correlations. While there is no reliable biomarker for choline consumption, fluctuations in choline intake per se may increase the variability in plasma concentrations of choline [36]. As an example, supplementation with krill oil, a rich source of PtdCho, has been associated with increasing plasma concentrations of choline as well as betaine [8]. Whole-grains and omega-3 fatty acids, both strongly represented in the dietary indexes of the present study, have previously shown positive associations with plasma betaine [10]. In contrast, non-processed meat, saturated fat and alcohol have displayed negative associations with plasma betaine in earlier studies [9].

The inverse relationships of tHcy and the diet index scores in our study are also supported by the literature. Fish, whole grains, and fruits and vegetables, again highly represented in our dietary indexes, have shown previous negative associations with tHcy concentrations [8, 9, 11]. Furthermore, observational studies in the U.S. and Netherlands have identified inverse associations between tHcy concentrations and intakes of choline, betaine and folate [12, 37]. One additional metabolite, serine, showed a positive association with the HNFI but not with the BSDS after adjustment for confounders. Interpretation of this result warrants caution since serine may also be synthesized endogenously from intermediates of glycolysis linking glucose metabolism with the choline oxidation pathway [38].

Higher intakes of B-vitamins could also partially explain the correlations observed in this study. Participants’ intakes of folate, vitamin B12, B2 and B6 all tended to be higher with increasing diet index score. Differences in folate consumption likely affect folate cycle activity, thereby impacting the rate of homocysteine re-methylation, which links directly to the conversion of betaine to DMG within the choline oxidation pathway. Other B-vitamins also function as co-factors to several reactions within one-carbon metabolism and therefore, intake of these vitamins may also have profound effects on metabolite concentrations [3, 39, 40].

Finally, a slightly weaker correlation was observed between the BSDS and plasma choline, compared to the HNFI and plasma choline. In previous studies, cholesterol has been shown to be positively associated with plasma choline concentrations [9]. However, increased meat consumption, a significant source of cholesterol, contributes to a lower BSDS score. Higher legume consumption, which gives a higher BSDS score, has been previously associated with lower plasma choline [9].

### Clinical relevance of the findings

The direction of beta coefficients were in line with the observed correlation coefficients even after adjustment for confounders. The predicted changes of metabolite concentrations were quite modest in comparison to previous studies. A study investigating plasma concentrations of choline and prostate cancer calculated relative risks based on a 100% increase in choline concentrations [37]. In a cross-sectional study analyzing the associations between one-carbon metabolites and colorectal cancer, significant decreases in odds ratio for high-risk adenomas were observed for plasma betaine increases of 50% or more [4]. For tHcy, plasma concentrations normally range from 5–15 µmol/L, while concentrations between 31–100 µmol/L, by convention, are designated as intermediately elevated and concentrations over 100 µmol/L as severely elevated [38].

### Intake of choline and betaine vs. the indexes

Overall, dietary intakes of choline and betaine showed significant linear correlations with both Nordic dietary indexes, which supports the positive associations between the indexes and observed plasma metabolite concentrations. The magnitude of these correlations ranged from low to moderate (*r* =  − 0.19 to 0.39). Positive correlations may be attributed to food items rich in these nutrients that are included in the HNFI and BSDS food sub-categories. Salmon, broccoli, beans and dairy products are major sources of choline, and these foods are well represented in the dietary indexes. The same is true for whole grains, which are considered major dietary sources of betaine [32, 41]. The addition of meat products and fat quality ratio to the BSDS may help explain why PtdCho did not show a positive relationship with this Nordic diet index score and why the choline form SM displayed a negative association. Red meat is an excellent source of both PtdCho and SM [32].

The positive correlation between dietary intakes of choline and betaine with the HNFI and BSDS indexes could rationally explain the positive associations of these indexes with blood concentrations of choline and betaine. However, further analysis of correlations between choline and betaine intakes and plasma metabolite concentrations could not confirm this. In fact, both nutrients showed slightly negative correlations with plasma choline and betaine highlighting the metabolic complexity of the choline oxidation pathway. These results also point to the possibility of other factors driving the positive associations between the Nordic dietary indexes and circulating choline oxidation metabolites. Similarly, the correlation coefficients between the different food components of the HNFI and BSDS indexes did not show positive associations with plasma choline and betaine. This indicates that the positive associations between the indexes and plasma choline and betaine are not simply the result of an additive effect of the separate food components. Other synergies or nutrient interactions within these dietary patterns could be at play. On the other hand, correlation coefficients between the food components of both the HNFI and BSDS and circulating tHcy appeared to be in-line with the negative association observed between index scores and tHcy.

### Strengths and limitations

To the best of our knowledge, this is the first study to investigate associations between a Nordic dietary pattern and the metabolites in the choline oxidation pathway. The population-based VIP provided a robust data set with close to 1,000 participants and included both dietary intake data and fasting blood samples for metabolite analysis. In addition, it was possible to perform detailed mapping of dietary intake parameters to the two separate healthy Nordic dietary indexes, making the study highly transparent and reproducible.

Limitations of the study included the use of a semi-quantitative FFQ, which are known to suffer from systematic errors such as over- and underreporting [23]. Another limitation is the fact that the FFQ had not been updated since its inception in 1984 to account for changes in consumption patterns in the Swedish population thus likely not covering the full diet of participants falling later in the study period, which spanned 17 years. With regards to the calculations of choline and betaine intake, it should be noted that the nutrient database used included only foods available in the U.S., which could affect the accuracy of these calculations.

The cross-sectional design of this study also limits the conclusions that can be drawn in terms of cause-and-effect relationship. Another limitation was that our data could not be stratified according to sex for separate analysis due to an insufficient number of observations. The sizes of correlation and regression coefficients, although statistically significant in several cases, were relatively small also limiting deeper interpretation of the results.

In conclusion, plasma concentrations of several metabolites involved in the choline oxidation pathway were associated with the healthy Nordic dietary indexes. Choline, betaine, tHcy and serine showed significant linear relationships with one or both indexes; however, the effect sizes were modest. Broadly speaking, higher adherence to a healthy Nordic diet corresponded to a more beneficial metabolic profile with increasing concentrations of betaine and decreasing concentrations of tHcy. While positive associations with plasma choline could be interpreted as an unhealthy attribute, it is important to note that the existing literature is inconclusive on associations between choline concentrations in the blood and increased disease risk. In addition, the plasma choline concentrations of this sample population, even participants with the highest dietary index scores, were well below concentration levels linked to increased cardiometabolic risk [7]. Further research is warranted to explore the underlying mechanisms involved in the associations between a healthy Nordic diet and these metabolites and with health outcomes in the target populations.

## Supplementary Information

**Additional file 1: Supplementary Table 1.** Food categories within the Healthy Nordic Food Index (HNFI), consumption data segmented by index scores. **Supplementary Table 2.** Food categories within the Baltic Sea Diet Score (BSDS), consumption data segmented by index scores. **Supplementary Figure 1. **Pearson correlation coefficients. Dietary choline and betaine intake (g/d*1000 kcal^-1^) vs. plasma metabolite concentrations. **Supplementary Figure 2. **Pearson correlation coefficients. HNFI food components (g/d*1000 kcal^-1^) vs. plasma metabolite concentrations. **Supplementary Figure 3. **Pearson correlation coefficients. BSDS food components (g/d*1000 kcal^-1^) vs. plasma metabolite concentrations.

## Data Availability

Data cannot be made freely available as they are subject to secrecy in accordance with the Swedish Public Access to Information and Secrecy Act [Offentlighets- och sekretesslagen, OSL, 2009:400], but can be made available to researchers upon request (subject to a review of secrecy). Requests for data should be made to Anna Winkvist, anna.winkvist@umu.se, or directed to Ingvar Bergdahl, director of the Biobank Research Unit, Umeå University, Sweden, ebf@umu.se.

## Abbreviations

24-HDR: 24-Hour dietary recall
BSDS: Baltic Sea Diet Score
BMI: Body mass index
CVD: Cardiovascular disease
DMG: Dimethylglycine
FFQ: Food frequency questionnaire
GPC: Glycerophosphocholine
HNFI: Healthy Nordic Food Index
PtdCho: Phosphatidylcholine
PCho: Phosphocholine
SM: Sphingomyelin
tHcy: Total homocysteine
USDA: U.S. Department of Agriculture
VIP: Västerbotten Intervention Programme
