# Supplementary material for: Healthy Nordic diet and associations with plasma concentrations of metabolites in the choline oxidation pathway: a cross-sectional study from Northern Sweden
Source: Nutr J. 2023 May 17;22:26. doi: 10.1186/s12937-023-00853-w (PMC10189923; doi:10.1186/s12937-023-00853-w)
Supplement: Supplementary file 1 — Additional file 1: Supplementary Table 1. Food categories within the Healthy Nordic Food Index (HNFI), consumption data segmented by index scores. Supplementary Table 2. Food categories within the Baltic Sea Diet Score (BSDS), consumption data segmented by index scores. Supplementary Figure 1. Pearson correlation coefficients. Dietary choline and betaine intake (g/d*1000 kcal-1) vs. plasma metabolite concentrations. Supplementary Figure 2. Pearson correlation coefficients. HNFI food components (g/d*1000 kcal-1) vs. plasma metabolite concentrations. Supplementary Figure 3. Pearson correlation coefficients. BSDS food components (g/d*1000 kcal-1) vs. plasma metabolite concentrations. [file 12937_2023_853_MOESM1_ESM.docx]

**Additional file**

**Supplementary Table 1.** Food categories within the Healthy Nordic Food Index (HNFI), consumption data segmented by index scores

|  |  | **HNFI segments** | | |
| --- | --- | --- | --- | --- |
|  | **All** | **1** | **2** | **3** |
| Observations, n | 969 | 386 | 411 | 172 |
| **Food category:^a, b^** |  |  |  |  |
| Fish  *grams/day*  *energy-adjusted* | 19 (12, 28)  11 (7, 16) | 14 (9, 21)  8 (5, 11) | 21 (14, 29)  12 (8, 18) | 25 (19, 36)  15 (11, 20) |
| Cabbages  *grams/day*  *energy-adjusted* | 11 (4, 26)  6 (2, 15) | 4 (1, 11)  3 (1, 6) | 15 (7, 29)  9 (4, 16) | 30 (17, 58)  19 (10, 34) |
| Whole grain rye  g*rams/day*  *energy-adjusted* | 56 (26, 78)  31 (18, 46) | 40 (21, 63)  22 (12, 35) | 58 (33, 88)  34 (21, 48) | 65 (55, 88)  40 (31, 53) |
| Whole grain oats  g*rams/day*  *energy-adjusted* | 17 (1, 72)  11 (0, 43) | 1 (0, 25)  1 (0, 15) | 28 (1, 73)  13 (1, 47) | 56 (22, 155)  36 (14, 82) |
| Apples and pears  g*rams/day*  *energy-adjusted* | 83 (37, 131)  47 (21, 87) | 41 (18, 83)  26 (11, 57) | 90 (42, 156)  54 (28, 100) | 122 (86, 230)  82 (53, 122) |
| Root vegetables  g*rams/day*  *energy-adjusted* | 18 (7, 37)  9 (3, 23) | 7 (3, 18)  4 (2, 9) | 18 (7, 50)  12 (5, 12) | 37 (27, 73)  27 (15, 41) |
| ^a^Values presented as medians and interquartile ranges.  ^b^Energy-adjusted values in grams/day*1000 kcal^-1^. | | | | |

**Supplementary Table 2.** Food categories within the Baltic Sea Diet Score (BSDS), consumption data segmented by index scores

|  |  | **BSDS segments** | | | | |
| --- | --- | --- | --- | --- | --- | --- |
|  | **All** | **1** | **2** | **3** | **4** | **5** |
| Observations, n | 969 | 131 | 240 | 170 | 226 | 202 |
| **Food category:^a, b^** |  |  |  |  |  |  |
| Fruits and berries  g*rams/day*  *energy-adjusted* | 90 (46, 161)  55 (26, 96) | 40 (21, 65)  22 (11, 44) | 63 (35, 115)  34 (21, 63) | 89 (46, 142)  57 (30, 95) | 120 (63, 180)  68 (39, 100) | 138 (104, 238)  100 (63, 145) |
| Vegetables  g*rams/day*  *energy-adjusted* | 67 (35, 116)  38 (19, 73) | 31 (14, 56)  18 (8, 33) | 45 (26, 84)  27 (15, 45) | 62 (38, 108)  39 (22, 70) | 78 (48, 129)  45 (27, 77) | 117 (78, 193)  76 (45, 118) |
| Cereals  g*rams/day*  *energy-adjusted* | 97 (56, 163)  58 (35, 94) | 48 (24, 86)  29 (15, 46) | 75 (45, 123)  43 (27, 63) | 88 (50, 125)  51 (35, 76) | 121 (82, 196)  69 (47, 111) | 155 (103, 238)  95 (66, 136) |
| Low-fat milk  g*rams/day*  *energy-adjusted* | 198 (76, 402)  125 (51, 219) | 59 (18, 198)  34 (11, 113) | 178 (47, 404)  102 (23, 209) | 209 (97, 402)  128 (66, 231) | 213 (136, 416)  130 (80, 223) | 269 (178, 452)  180 (118, 256) |
| Fish  g*rams/day*  *energy-adjusted* | 19 (12, 28)  11 (7, 16) | 14 (6, 21)  8 (4, 12) | 17 (9, 26)  10 (6, 13) | 17 (12, 25)  11 (7, 15) | 21 (14, 29)  12 (8, 17) | 22 (16, 35)  15 (9, 21) |
| Meat products  g*rams/day*  *energy-adjusted* | 73 (52, 100)  44 (33, 58) | 95 (65, 134)  57 (42, 73) | 81 (61, 116)  48 (37, 62) | 69 (50, 92)  43 (33, 56) | 71 (50, 88)  41 (31, 54) | 60 (45, 84)  38 (28, 48) |
| ^a^Values presented as medians and interquartile ranges.  ^b^Energy-adjusted values in grams/day*1000 kcal^-1^. | | | | | | |


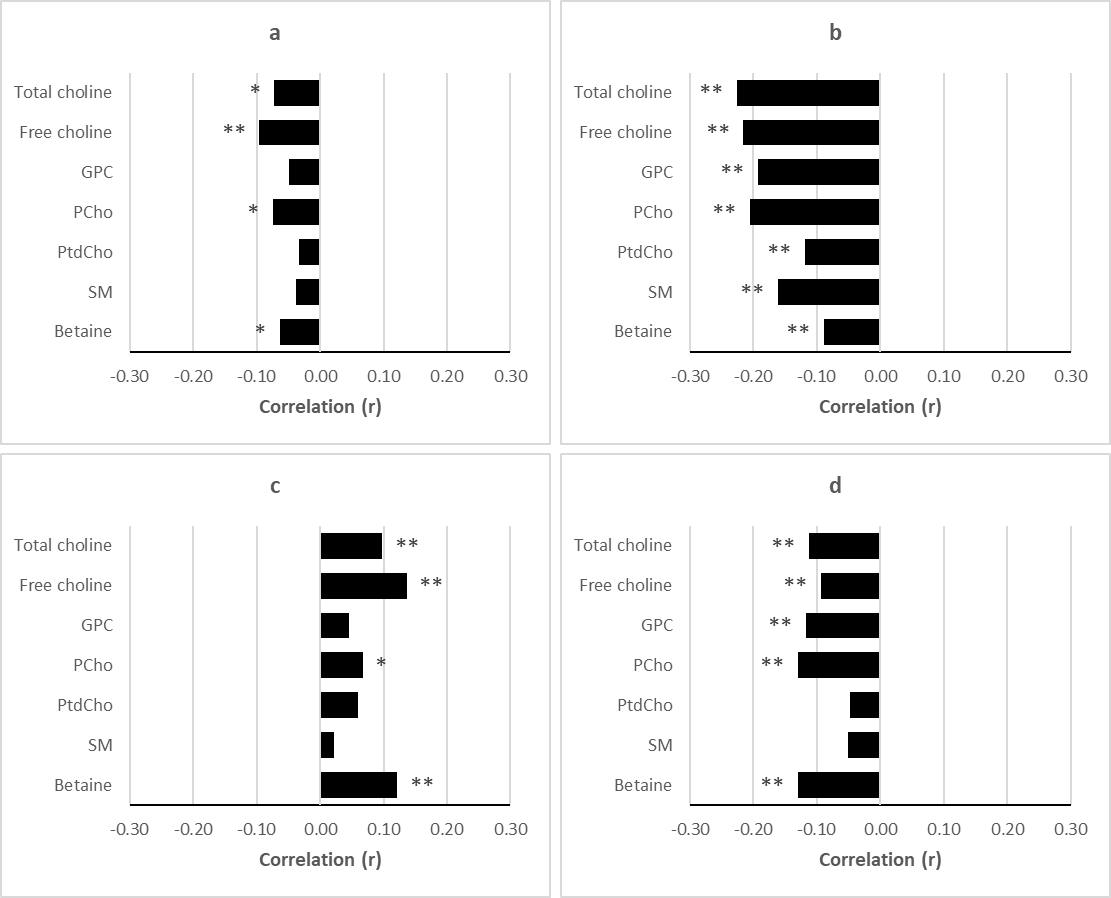


**Supplementary Figure 1.** Pearson correlation coefficients. Dietary choline and betaine intake (g/d*1000 kcal^-1^) vs. plasma metabolite concentrations.

Legend: a - Choline. b - Betaine. c - Serine. d - Total homocysteine. * P<0.05. ** P<0.01.


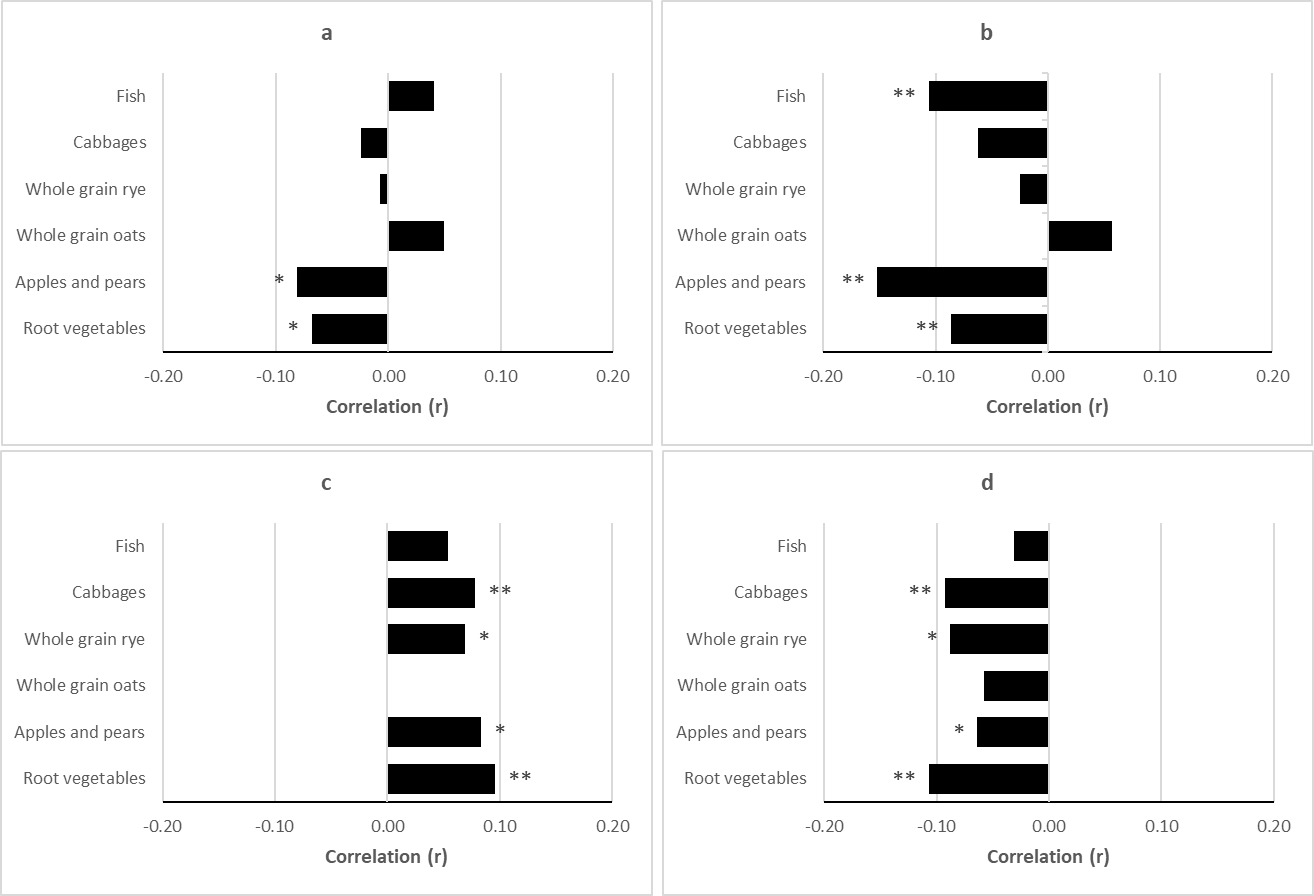


**Supplementary Figure 2.** Pearson correlation coefficients. HNFI food components (g/d*1000 kcal^-1^) vs. plasma metabolite concentrations.

Legend: a - Choline. b - Betaine. c - Serine. d - Total homocysteine. * P<0.05. ** P<0.01.


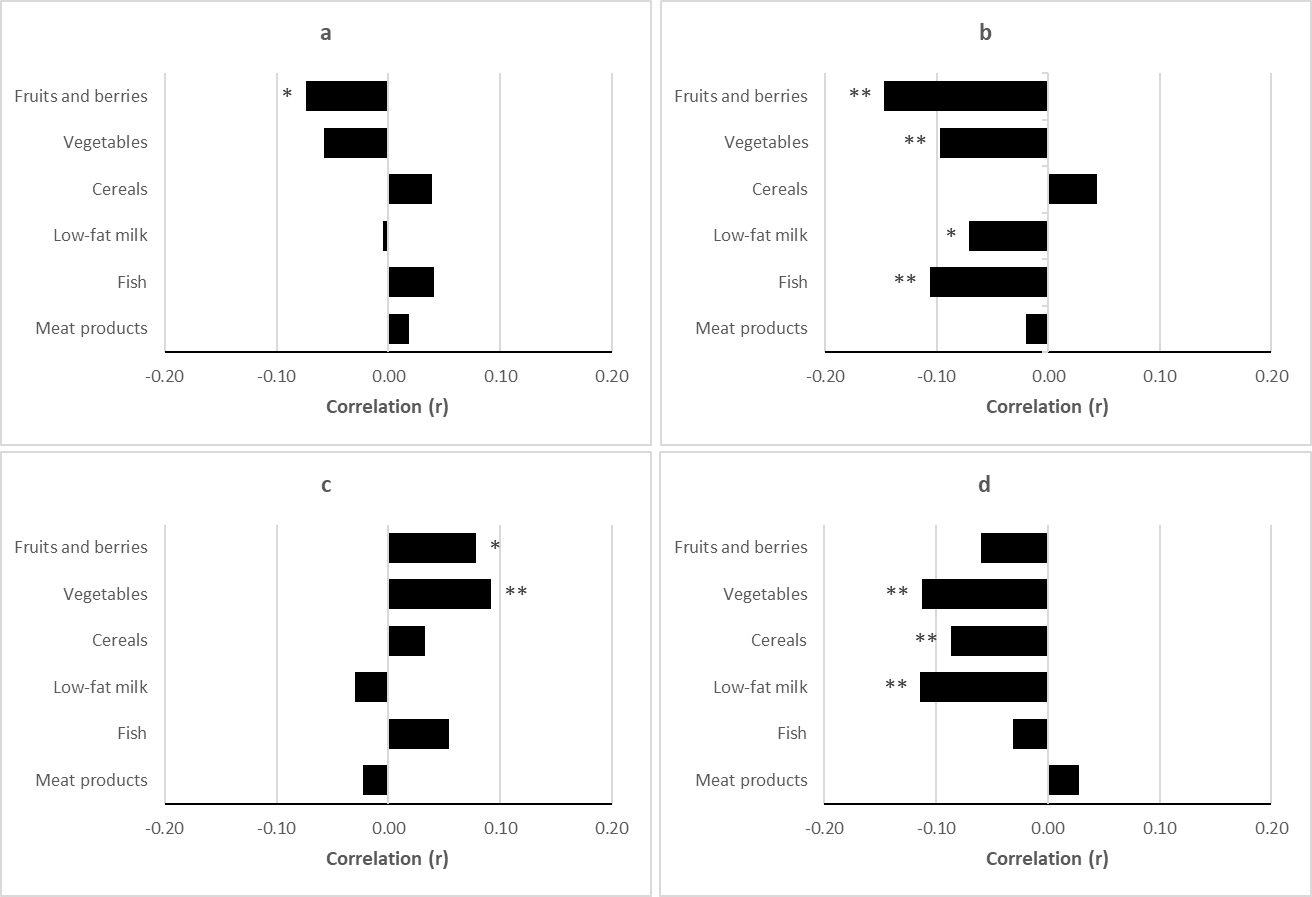


**Supplementary Figure 3.** Pearson correlation coefficients. BSDS food components (g/d*1000 kcal^-1^) vs. plasma metabolite concentrations.

Legend: a - Choline. b - Betaine. c - Serine. d - Total homocysteine. * P<0.05. ** P<0.01.
